# DnaA and LexA Proteins Regulate Transcription of the *uvrB* Gene in *Escherichia coli*: The Role of DnaA in the Control of the SOS Regulon

**DOI:** 10.3389/fmicb.2018.01212

**Published:** 2018-06-18

**Authors:** Wurihan, Gezi, Elisa Brambilla, Shuwen Wang, Hongwei Sun, Lifei Fan, Yixin Shi, Bianca Sclavi, Morigen

**Affiliations:** ^1^State Key Laboratory of Reproductive Regulation and Breeding of Grassland Livestock, School of Life Sciences, Inner Mongolia University, Hohhot, China; ^2^LBPA, UMR 8113, CNRS, ENS Paris-Saclay, Cachan, France; ^3^School of Life Sciences, Arizona State University, Tempe, AZ, United States

**Keywords:** DnaA, LexA, *uvrB* gene expression, SOS regulon, regulation

## Abstract

The *uvrB* gene belongs to the SOS network, encoding a key component of the nucleotide excision repair. The *uvrB* promoter region contains three identified promoters with four LexA binding sites, one consensus and six potential DnaA binding sites. A more than threefold increase in transcription of the chromosomal *uvrB* gene is observed in both the Δ*lexA* Δ*sulA* cells and *dnaA*_A345S_ cells, and a fivefold increase in the Δ*lexA* Δ*sulA dnaA*_A345S_ cells relative to the wild-type cells. The full activity of the *uvrB* promoter region requires both the *uvrB*p1-2 and *uvrB*p3 promoters and is repressed by both the DnaA and LexA proteins. LexA binds tightly to LexA-box1 at the *uvrB*p1-2 promoter irrespective of the presence of DnaA and this binding is important for the control of the *uvrB*p1-2 promoter. DnaA and LexA, however, compete for binding to and regulation of the *uvrB*p3 promoter in which the DnaA-box6 overlaps with LexA-box4. The transcription control of *uvrB*p3 largely depends on DnaA-box6. Transcription of other SOS regulon genes, such as *recN* and *dinJ*, is also repressed by both DnaA and LexA. Interestingly, the absence of LexA in the presence of the DnaA_A345S_ mutant leads to production of elongated cells with incomplete replication, aberrant nucleoids and slow growth. We propose that DnaA is a modulator for maintenance of genome integrity during the SOS response by limiting the expression of the SOS regulon.

## Introduction

The *uvrB* gene encodes the UvrB protein, one of the key components of the NER system (Truglio et al., 2006). NER repair is a versatile pathway that recognizes a wide range of DNA lesions by the concerted function of the UvrABC proteins (Pruteanu and Baker, 2009). The *uvrB* gene belongs to the SOS regulon (Howard-Flanders et al., 1966). SOS is a global response to DNA damage in which RecA filaments bound on ssDNA promote self-cleavage of the LexA protein. Cleavage of LexA induces expression of the SOS genes, resulting in an arrest of cell division for the time required to repair the damages (Walter, 1996). LexA regulates the SOS regulon by binding to the LexA-box and thus preventing gene expression during normal growth. The LexA-box has the following consensus sequence TACTG(TA)_5_CAGTA (Walker, 1984), having a conserved trimer of CTG on the left and another trimer of CAG on the right with a variable sequence of spacers; the spacing between “CTG” and “CAG” is invariable at 10 nucleotides (Fernandez De Henestrosa et al., 2000; Wade et al., 2005). LexA contains two domains: an N-terminal winged helix-turn-helix (wHTH) DNA-binding domain and a C-terminal dimer with a latent protease domain (Zhang et al., 2010). In response to DNA damage RecA-ssDNA-ATP filaments are formed and the auto-proteolytic activity of LexA at the C-terminal domain is activated by interacting with the filaments. The degradation of LexA opens the promoter region for RNA polymerase (RNAP) recruitment and the start of transcription.

The DnaA protein initiates chromosomal replication in bacteria by interacting with 9-mer consensus sequences of TTA/TTNCACA, the DnaA-boxes, at the origin for replication (Kornberg and Baker, 1992; Schaper and Messer, 1995). DnaA has a high affinity for ATP and ADP (Sekimizu et al., 1987), and ATP-DnaA is active for the initiation of replication whereas ADP-DnaA is inactive (Sekimizu et al., 1987). DnaA is also a transcription factor, repressing transcription by binding to DnaA-boxes in the promoter regions, such as those found at the promoters for the *dnaA* gene itself (Atlung et al., 1985; Braun et al., 1985), the *mioC* gene (Lother et al., 1985) and the *nrd* operon (Tuggle and Fuchs, 1986; Speck et al., 1999; Olliver et al., 2010) while transcription of the *polA* gene is stimulated by DnaA in stationary phase (Quinones et al., 1997).

The *E. coli uvrB* promoter region contains three promoters, namely, *uvrB*p1, *uvrB*p2, and *uvrB*p3 (Sancar et al., 1982). A LexA-box with the AACTGTTTTTTTATCCAGTA sequence has been identified between the -35 and -10 regions of *uvrB*p2 (Fernandez De Henestrosa et al., 2000). Interestingly, the *uvrB*p3 promoter contains DnaA boxes (Arikan et al., 1986) which constitute the DARS1 (DnaA reactivation site) site consisting of three DnaA-boxes, where inactive ADP-DnaA is reactivated to form active ATP-DnaA (Fujimitsu et al., 2009). Here, we show that the *uvrB* promoters are repressed by both DnaA and LexA by specifically binding to its promoter region in either a competitive or an independent manner. Interestingly, two other genes of the SOS regulon, *recN* and *dinJ*, are also found to be repressed by both DnaA and LexA. The simultaneous absence of LexA- and DnaA-dependent repression leads to production of elongated cells with incomplete DNA replication with abnormal nucleoids and slow growth. It is likely that regulation of gene expression by DnaA maintains genome integrity during the SOS response in *Escherichia coli.*

## Materials and Methods

### Bacterial Strains

All bacterial strains used in this study are derived from the *E. coli* K12 listed in **Table 1**. The Δ*sulA*::*neo* allele was transferred into the MC4100 by P1 transduction (Miller et al., 1992) and resulting in MC4100Δ*sulA*::*neo*. The *cat* gene was PCR amplified using the plasmid pKD3 as template and primer 582 and 583 as listed in Supplementary Table S2 and inserted into MC4100Δ*sulA*::*neo* mutant to replace the chromosomal *lexA* gene through homologous recombination by One-step Chromosomal Gene Inactivation method (Datsenko and Wanner, 2000), resulting in MC4100Δ*sulA*::*neo*Δ*lexA*::*cat* double mutant. The *neo* or/and *cat* genes were removed by the FRT site-specific recombination as described previously (Datsenko and Wanner, 2000), resulting in MC4100Δ*sulA* or/and MC4100Δ*sulA*Δ*lexA* double mutant. The *cat* gene was also PCR amplified using pKD3 as template and primers 48 and 49 listed in Supplementary Table S2, then inserted behind the chromosomal *uvrB* gene in MC4100 and MC4100Δ*sulA*Δ*lexA* cells by the method mentioned above. The *cat* gene was replaced by pCE36 using the FRT site-specific recombination in the cells mentioned, resulting in insertion of the *lacZ*…*neo* fusion behind the chromosomal *uvrB* gene as described previously (Datsenko and Wanner, 2000; Ellermeier et al., 2002). As a result, MC4100*uvrB-lacZ*…*neo*, MC4100Δ*sulA uvrB-lacZ*…*neo* and MC4100Δ*sulA*Δ*lexA uvrB-lacZ*…*neo* cells were constructed. The *dnaA*_A345S_…*cat* allele was transferred into the MC4100*uvrB-lacZ*…*neo*, MC4100Δ*sulA uvrB-lacZ*…*neo* and MC4100Δ*sulA*Δ*lexA uvrB-lacZ*…*neo* cells by P1 transduction (Miller et al., 1992). The *lexA*3…*tet* allele was P1 transduced into MC4100 and *dnaA*_A345S_…*cat* cells carrying *uvrB-lacZ*…*neo* fusion, respectively (Miller et al., 1992). For construction of *recN*-*lacZ*…*neo* and *dinJ*-*lacZ*…*neo*, the *cat* gene was PCR amplified using the plasmid pKD3 as template and primers 1229 and 1230 for *recN*-*lacZ*…*neo*, primers 1235 and 1236 for *dinJ*-*lacZ*…*neo* as listed in Supplementary Table S2. After insertion of the *cat* gene down-stream of the chromosomal *recN or dinJ* gene in MC4100 cells, the *cat* gene was replaced by pCE36 using the FRT site-specific recombination, resulting in insertion of the *lacZ*…*neo* fusion behind the chromosomal *recN or dinJ* gene as described previously (Datsenko and Wanner, 2000; Ellermeier et al., 2002). The *recN*-*lacZ*…*neo or dinJ*-*lacZ*…*neo* allele was P1 transduced into *dnaA*_A345S_, Δ*sulA*Δ*lexA*, Δ*sulA*Δ*lexA dnaA*_A345S_ and *lexA*3 *dnaA*_A345S_ cells, resulting in *dnaA*_A345S_ *recN-lacZ*…*neo*, Δ*sulA*Δ*lexA recN-lacZ*…*neo*, Δ*sulA*Δ*lexA dnaA*_A345S_ *recN-lacZ*…*neo*, *lexA*3 *dnaA*_A345S_ *recN-lacZ*…*neo*, or *dnaA*_A345S_ *dinJ-lacZ*…*neo*, Δ*sulA*Δ*lexA dinJ-lacZ*…*neo*, Δ*sulA*Δ*lexA dnaA*_A345S_ *dinJ-lacZ*…*neo* and *lexA*3 *dnaA*_A345S_ *dinJ-lacZ*…*neo*. DH5α was used as a host for the preparation of plasmid DNA. The WM2287 strain containing the p*dnaA*116 plasmid was used for DnaA purification (Schaper and Messer, 1995) and BL21-Gold (DE3) for His_6_-LexA protein expression and purification.

**Table 1:** Bacterial strains.

Strain	Genotype	Source
MC4100	Wild type F^-^*araD*139Δ (*lac*) U169 *strA thi*	Casadaban, 1976; Ferenci et al., 2009
BW25113	Wild type *rrnB*3 Δ*lacZ*4787 *hsdR*514 Δ(*araBAD*)567Δ (*rhaBAD*)568 *rph-1*	Baba et al., 2006
JW0941-KC	BW25113Δ *sulA*::*neo*	Baba et al., 2006
SMG379	MG1655 *dnaA*_A345S_…MiniTn10 *cat*	Gon et al., 2006
JC13199	*lexA3malE...*Tn10	Clark, 1973
MOR741	MC4100 *uvrB-lacZ*…*neo*	This work
MOR746	MC4100 *dnaA*_A345S_…*cat uvrB-lacZ*…*neo*	This work
MOR2395	MC4100 Δ*sulA*Δ*lexA uvrB*-*lacZ*… *neo*	This work
MOR2399	MC4100 Δ*sulA* Δ*lexA dnaA*_A345S_…*cat uvrB-lacZ*…*neo*	This work
MOR2670	MC4100 Δ*sulA*::*neo*	This work
MOR2672	MC4100 Δ*sulA uvrB*-*lacZ*… *neo*	This work
MOR798	MC4100 *lexA*3…*tet uvrB-lacZ*…*neo*	This work
MOR803	MC4100 *lexA*3… *tet dnaA*_A345S_…*cat uvrB-lacZ*…*neo*	This work
MOR749	MC4100 *dnaA*_A345S_…*cat*	This work
MOR1466	MC4100 Δ*sulA*Δ*lexA*	This work
MOR1511	MC4100 Δ*sulA*Δ*lexA dnaA*_A345S_…*cat*	This work
MOR2585	MC4100 *recN-lacZ*…*neo*	This work
MOR2586	MC4100 *dnaA*_A345S_…*cat recN-lacZ*…*neo*	This work
MOR2587	MC4100 Δ*sulA*Δ*lexA recN*-*lacZ*… *neo*	This work
MOR2588	MC4100 Δ*sulA* Δ*lexA dnaA*_A345S_…*cat recN-lacZ*…*neo*	This work
MOR2589	MC4100 *lexA*3…*tet dnaA*_A345S_…*cat recN-lacZ*…*neo*	This work
MOR2590	MC4100 *dinJ-lacZ*…*neo*	This work
MOR2591	MC4100 *dnaA*_A345S_…*cat dinJ-lacZ*…*neo*	This work
MOR2592	MC4100 Δ*sulA*Δ*lexA dinJ*-*lacZ*… *neo*	This work
MOR2593	MC4100 Δ*sulA* Δ*lexA dnaA*_A345S_…*cat dinJ-lacZ*…*neo*	This work
MOR2594	MC4100 *lexA*3…*tet dnaA*_A345S_…*cat dinJ-lacZ*…*neo*	This work
BL21-Gold (DE3)	*E. coli* B F^-^*ompT hsdS*B (r_B_^-^m_B_^-^) *dcm^+^* Tet^r^ *gal* (DE3) *endA* Hte	Agilent Technologies
DH5α	F^-^ *sup*E44 *Δlac*U169(*ΔlacZΔM15*) *hsdR*17 *recA*1 *endA*1 *gyrA*96	New England Biolabs

### Growth Media and Conditions

Cells were grown at 37°C in LB (Bertani, 1951) or ABTGcasa medium (Morigen et al., 2005). When necessary, 100 μg/ml of ampicillin, 30 μg/ml of chloramphenicol, 15 μg/ml of tetracycline and 50 μg/ml of kanamycin were added.

### Plasmid Constructions

All plasmids used in this study are listed in **Table 2** and the primers including their descriptions are listed in Supplementary Table S2. The *uvrB*p1-3 promoter was PCR amplified using chromosomal DNA from the wild-type BW25113 cells as template and primers 54 and 57. The *uvrB*p1-3 PCR fragment was inserted in front of the promoterless *lacZ* gene on pTAC3953 (Brondsted and Atlung, 1994) at *Bam*HI and *Hin*dIII sites, resulting in plasmid p*uvrB*p1-3-*lacZ*. Using the same template, the *uvrB*p1-2 promoter region was amplified by primers 79 and 57, and the *uvrB*p3 promoter region by primers 54 and 71. The PCR fragment for each promoter was then inserted into pTAC3953 at the *Bam*HI and *Hin*dIII sites (Brondsted and Atlung, 1994), leading to the construction of plasmids p*uvrB*p1-2-*lacZ* and p*uvrB*p3-*lacZ*. The *uvrB*p1-3-*lacZ* fusion was PCR amplified by primers 54 and 1131 using p*uvrB*p1-3-*lacZ* as template. The PCR fragment was then inserted into a low copy plasmid, MiniR1 which is about 1–2 copies per the chromosomal *ter* site (Morigen et al., 2001) at the *Bam*HI and *Hin*dIII sites, resulting in MiniR1-*uvrB*p1-3-*lacZ* (shown as R1-*uvrB*p1-3 for short). The *uvrB*p1-2-*lacZ* fusion was PCR amplified by primers 79 and 1350 using p*uvrB*p1-2-*lacZ* as template. The PCR fragment was then inserted into MiniR1 at the *Bam*HI and *Bgl*II sites, resulting in MiniR1-*uvrB*p1-2-*lacZ* (R1-*uvrB*p1-2 for short). The *uvrB*p3-*lacZ* fusion was PCR amplified by primers 54 and 1350 using p*uvrB*p3-*lacZ* as a template. The PCR fragment was inserted into MiniR1 at *Bam*HI and *Bgl*II sites, resulting in MiniR1-*uvrB*p3-*lacZ* (R1-*uvrB*p3 for short). The DH5α cells were transformed with the resulting ligation. The *lexA* gene was PCR amplified using the chromosomal DNA from the wild-type BW25113 cells as template and primers 578 and 579. The PCR fragment for *lexA* was inserted into pET28a (EMD Biosciences) at the *Nco*I and *Xho*I sites, resulting in pET28a-his_6_-*lexA* which produces His_6_-LexA protein fusion under IPTG induction. All constructions were sequenced to make sure the plasmid constructions were correct.

**Table 2:** Plasmids.

Plasmids	Description	Source
pKD3	*rep*_R6K_ *bla* FRT *cat* FRT	Datsenko and Wanner, 2000
pKD4	*rep*_R6K_ *bla* FRT *neo* FRT	Datsenko and Wanner, 2000
pKD46	*rep*_pSC101_^ts^*bla* P_araBAD_ γβ exo	Datsenko and Wanner, 2000
pCP20	*rep*_pSC101_^ts^ *bla cat cI*857P_R_	Datsenko and Wanner, 2000
pCE36	*rep*_R6K_ *neo* FRT *lacZY* t*_his_*	Ellermeier et al., 2002
pET-28a	*rep*_ColE1_ *neo lacI* P_T7_	EMD Biosciences
pET28a-his_6_-*lexA*	The *lexA* gene was inserted into pET28a at *Nco*I and *Xho*I to produce His_6_-LexA fusion.	This work
p*dnaA*116	*rep*_ColE1_ *bla lacI^+^*P_A1-03/04_ *dnaA ter_trpA_*	Krause et al., 1997
pTAC3953	*rep*_Pmd_ *neo lacZ*	Brondsted and Atlung, 1994
p*uvrB*p1-3-*lacZ*	The whole cluster of the *uvrB* promoters including *uvrB*p1, 2, and 3 was inserted in front of the *lacZ* gene on pTAC3953 at the *Bam*HI and *Hin*dIII sites.	This work
p*uvrB*p1-2-*lacZ*	The *uvrB*p1-2 fragment was inserted in front of the *lacZ* gene on pTAC3953 at the *Bam*HI and *Hin*dIII sites.	This work
p*uvrB*p3-*lacZ*	The *uvrB*p3 fragment was inserted in front of the *lacZ* gene on pTAC3953 at the *Bam*HI and *Hin*dIII sites.	This work
p*uvrB*pΔ279-172-*lacZ*	A fragment from -279 to -172 was deleted from the plasmid p*uvrB*p1-3-*lacZ.*	This work
MiniR1	R1 derived vector, containing *oriR*1, *bla*, *repA*, *tap*, *copA*	Morigen et al., 2001
MiniR1-*uvrB*p1-3-*lacZ*	The *uvrB*p1-3-*lacZ* fusion fragment was inserted at the *Bam*HI and *Hin*dIII sites onto MiniR1.	This work
MiniR1-*uvrB*p1-2-*lacZ*	The *uvrB*p1-2-*lacZ* fragment was inserted at the *Bam*HI and *Bgl*II sites onto MiniR1.	This work
MiniR1-*uvrB*p3-*lacZ*	The *uvrB*p3-*lacZ* fragment was inserted at the *Bam*HI and *Bgl*II sites onto MiniR1.	This work
p*uvrB*p1-2GC-*lacZ*	TG in LexA-box1 was replaced by GC on p*uvrB*p1-2-*lacZ.*	This work
p*uvrB*p3CA-*lacZ*	TG in DnaA-box6 was replaced by CA on p*uvrB*p3-*lacZ.*	This work

### Site-Directed Mutagenesis

Point mutation was generated using a site-directed mutagenesis kit (TransGen Biotech, Beijing, China) as described previously (Rousseau et al., 2013). The mutations (from TG to GC) in LexA-box1 in the *uvrB*p1-2 promoter were generated by site-directed mutagenesis using the p*uvrB*p1-2-*lacZ* plasmid as template and the pair of primers 1214 and 1215. Similarly, using the p*uvrB*p3 plasmid as template, the mutations (from TG to CA) in DnaA-box6 in *uvrB*p3 promoter, were generated by the pair of primers 1210 and 1211.

### Purification of Proteins

The DnaA protein was over-expressed in WM2287/p*dnaA*116 cells (Krause et al., 1997) and purified as described previously (Olliver et al., 2010). The BL21-Gold (DE3)/pET28a-his_6_-*lexA* cells were grown at 37°C in 200 ml of LB medium. At OD_600_ = 0.6, IPTG with 0.3 mM of final concentration was added and incubated for 2 h. The cells were harvested and washed with PBS once, resuspended in 10 ml of the lysis buffer (50 mM NaH_2_PO_4_, 300 mM NaCl, 10 mM imidazole), and sonicated. The whole cell lysate was mixed with His-Select Ni-NTA slurry (Qiagen) and His_6_-LexA was purified according to the manufacturer instructions. Purity of the His_6_-LexA protein sample was detected by staining with INSTANT BLUE (Expedeon) after SDS-PAGE (Supplementary Figure S1) gel-electrophoresis following the manufacturer instructions. The His_6_-LexA protein concentration was determined by BCA assay (Thermo Scientific) and stored at -80°C after imidazole was removed by dialysis in 1xPBS buffer (0.137 M NaCl, 0.027 M KCl, 0.01 M Na_2_HPO_4_, 0.0018 M KH_2_PO_4_).

### β-Galactosidase Activity Assay

Exponentially growing cells (1 ml) at 37°C in ABTGcasa medium were collected at OD_450_ = 0.1, 0.2, 0.3, 0.4, and 0.5, then mixed with cold toluene (0.1 ml) and kept on ice immediately. For measurement of β-galactosidase activity, 0.2 ml toluene-treated sample was added to 1 ml Z buffer (40 mM NaH_2_PO_4_, 60 mM Na_2_HPO_4_, 10 mM KCl, 1 mM MgSO_4_ and 50 mM β-mercaptoethanol, pH 7.0) containing 0.66 mg/ml *o*-nitrophenyl-β-D-galactopyranoside. The reaction was performed at 30°C until the color changed to yellow and stopped by addition of 0.5 ml 1 M Na_2_CO_3_, and the absorbance at OD_420_ was measured. The β-galactosidase activity was calculated by 1000^∗^OD_420_/reaction time (min) ^∗^OD_450_^∗^0.2 ml (Miller, 1972).

### DNase I Footprinting Assays

The *uvrB* promoter region (523bp) was PCR amplified using chromosomal DNA from the wild-type BW25113 cells as template and primer 828 and 829 for the DNase I footprinting assays. In the PCR reaction, the primer 828 was 5′ labeled with [γ-^32^P] ATP (GE Healthcare) by T4 polynucleotide kinase (New England Biolabs). The PCR product was purified with the Bio-Spin 6 Columns (Bio-Rad) according to the manufacturer instructions. Approximately 1 nmol of labeled DNA and increasing amounts (final concentration was 50, 100, 200, 300, and 600 nM) of ATP-DnaA or His_6_-LexA protein were mixed in a 10 μl reaction buffer containing 1 mM DTT, 0.5 mg/ml Ac-BSA, 20 mM HEPES pH 7.6, 50 mM *K*-glu, 5 mM MgCl_2_ and 3 mM of ATP (Sigma-Aldrich). The reaction mixture was incubated at 37°C for 15 min. Then, 4 mg/ml of DNase I diluted in digestion buffer (25 mM Tris pH 7.5, mM MgCl_2_, 1 mM CaCl_2_, 2 mM DTT and 100 mM KCl) was added and the mixture was incubated at 37°C for 20 s. To determine the protection patterns of the *uvrB* promoter DNA by two proteins (DnaA and LexA), the second protein was added with final concentrations of 50, 100, 200, 300, and 600 nM after 10 min incubation with the first protein with final concentration of 200 nM, and incubated for 10 min, then digested by DNase I for 30 s. The DNase I digestion was stopped by addition of an equal volume of formamide loading buffer (90% formamide, 1 × TBE, bromophenol blue, xylene cyanol, calf thymus non-specific DNA). Samples were incubated for 5 min at 95°C and analyzed by 6% acrylamide in denaturing conditions (8 M urea and 1 × TBE buffer) by comparison with a DNA sequence ladder generated with the same primers using a A+G reaction as described previously (Maxam and Gilbert, 1980). After electrophoresis, gels were dried and autoradiographed.

### UV Irradiation

Cells were exponentially grown at 37°C in 50 ml of ABTGcasa medium, 20 ml of cell culture at OD_450_ = 0.08 was irradiated in an open petri-dish with 50 J/M^2^ of UV, then cells were grown in flask at 37°C. Sampling and measurement of β-galactosidase activity was carried out as mentioned above.

### Flow Cytometry

Exponentially growing cells in ABTGcasa medium at 37°C were treated with 300 μg/ml rifampicin and 10 μg/ml cephalexin for 4–5 generations. Initiation of DNA replication is inhibited by rifampicin which allows ongoing replication finish while cell division is blocked by cephalexin at the time of addition of the drugs (Skarstad et al., 1986; Boye and Lobner-Olesen, 1991). Cells were fixed in 70% ethanol and stained in Hoechst 33258 (Invitrogen) for 30 min, then analyzed by flow cytometry (LSR Fortessa, BD).

### Confocal Fluorescence Microscopy

Exponentially growing cells in ABTGcasa medium at 37°C were harvested, fixed in 70% ethanol, visualized under a Zeiss LSM710 Confocal microscope with 100×/1.4 Plam-Apo at 405 nm laser excitation after staining in Hoechst 33258 for 30 min. Images were scanned by a PMP detector and analyzed with the ZEN 2011 (black version) software to measure cell size and nucleoid distribution.

## Results

### Both DnaA and LexA Repress Expression of the *uvrB* Gene

**FIGURE 1:**
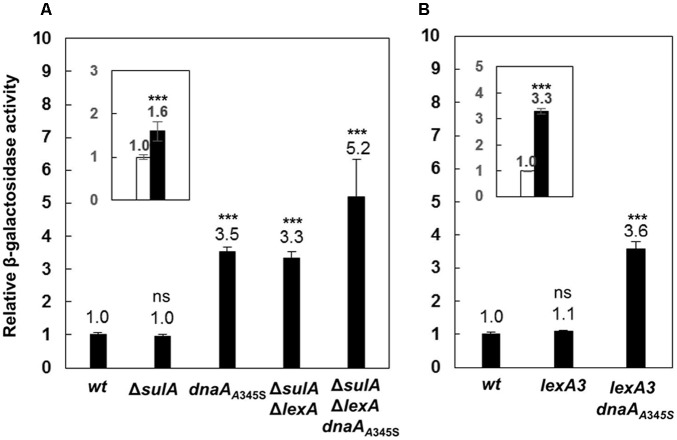
The DnaA and LexA proteins repress expression of the *uvrB* gene independently. **(A)** The wild-type, Δ*sulA, dnaA*_A345S_, Δ*sulA*Δ*lexA* and Δ*sulA*Δ*lexA dnaA*_A345S_ cells carrying *uvrB*-*lacZ* fusion on their chromosomes were exponentially grown in ABTGcasa medium at 37°C. The β-galactosidase activity from *uvrB*-*lacZ* fusion in the cells was determined by Miller method (Miller, 1972), and relative expressions of *uvrB-lacZ* in all mutants to that in the wild-type (14 U) were calculated. The inset indicates the relative expression of *uvrB-lacZ* in the Δ*sulA*Δ*lexA dnaA*_A345S_ cells against that in the Δ*sulA*Δ*lexA* cells. **(B)** The β-galactosidase activity in exponentially growing *lexA*3 and *lexA*3*dnaA*_A345S_ cells carrying *uvrB*-*lacZ* fusion was determined as described in the legend to **(A)**. The inset indicates the relative expression of *uvrB-lacZ* in the *lexA*3*dnaA*_A345S_ cells against that in the *lexA*3 cells. The values shown at top of the bars are the average of three individual experiments, and the standard errors are shown. ^∗∗∗^Stands for *P*-value ≤ 0.01, ^∗∗^for 0.01 < *P*-value ≤ 0.05, and ns represents *P*-value > 0.05.

A global transcriptional analysis by using Affymetrix GeneChip *E. coli* Genome 2.0 arrays showed that expression of the *uvrB* gene increased 2.7 (±0.9)-fold in a *dnaA*_A345S_ mutant relative to the wild-type cells (Morigen and Skarstad, unpublished data). The result suggests that DnaA could directly be involved in control of the *uvrB* gene expression since DnaA_A345S_ binds to DnaA-boxes with a lower affinity compared to wild-type DnaA. The *dnaA*_A345S_ mutant is a suppressor for a mutant lacking four of the redoxins involved in Nrd activity (Ortenberg et al., 2004) and the purified DnaA_A345S_ protein is defective for ATP binding *in vitro* (Gon et al., 2006). The *dnaA*_A345S_ mutant is also found to result in under-replication and larger cell mass with slower growth (Ortenberg et al., 2004; Gon et al., 2006). In order to determine the regulatory effect of DnaA on transcription of the *uvrB* gene, the *lacZ* reporter gene was inserted downstream of the chromosomal *uvrB* gene, resulting in an *uvrB-lacZ* derivative of the MC4100 strain lacking the chromosomal *lacZ* gene (Casadaban, 1976; Ferenci et al., 2009). Subsequently, a *dnaA*_A345S_ allele was transferred to the *uvrB-lacZ* strain by P1 transduction. The *uvrB* gene belongs to the SOS regulon which is regulated by LexA (Howard-Flanders et al., 1966). It should be noted that the LexA protein is essential for cell growth but the growth defect in the absence of LexA can be suppressed by deletion of the *sulA* gene (George et al., 1975). Thus, we removed the *lexA* gene by constructing the Δ*lexA* Δ*sulA* double mutant. To understand how DnaA interacts with LexA in the control of *uvrB* expression, the *uvrB-lacZ* allele was P1 transduced into the Δ*lexA* Δ*sulA*, Δ*lexA* Δ*sulA dnaA*_A345S_, *lexA3* and *lexA3 dnaA*_A345S_ cells including the Δ*sulA* mutant as a control. The level of transcription from the *uvrB* promoter region was measured by the β-galactosidase activity assay in exponentially growing cells. Transcription from the *uvrB* promoter was 3.5-fold higher in the *dnaA*_A345S_ cells compared with that of the wild-type cells (**Figure 1A**), suggesting that DnaA represses *uvrB* expression. Not surprisingly, transcription from the *uvrB* promoter region in the Δ*sulA* mutant was about the same as that in the wild-type cells, while it was 3.3-fold higher in the Δ*lexA*Δ*sulA* mutant compared to the control, in agreement with previous work (Sancar et al., 1982), indicating that LexA represses *uvrB* expression. Interestingly, *uvrB* transcription was further increased, to 5.2-fold, in the Δ*lexA*Δ*sulA dnaA*_A345S_ triple mutant (**Figure 1A**), implying that the repression by DnaA and LexA of *uvrB* expression is additive and thus might be independent of an interaction between the two proteins. The conclusion is supported by 1.6-fold increase of *uvrB* expression in the Δ*sulA*Δ*lexA dnaA*_A345S_ cells compared to that in the Δ*sulA*Δ*lexA* cells (**Figure 1A** inset). The LexA3 mutant protein is not self-cleavable or largely resistant to cleavage and thus binds to the *uvrB* promoter region regardless of whether the SOS response is on or off (Little et al., 1980; Markham et al., 1981). Transcription from the *uvrB* promoter region in the *lexA*3 mutant was about the same as that in the wild-type cells, while it was 3.6-fold higher in the *lexA*3 *dnaA*_A345S_ strain (**Figure 1B**). These results support the idea that DnaA-dependent repression of *uvrB* expression is independent of LexA activity. We conclude that in the absence of the SOS response both DnaA and LexA repress transcription of the *uvrB* gene and function independently of each other.

### The *uvrB* Promoter Region Contains Seven Potential DnaA-Boxes and Four LexA-Boxes

**FIGURE 2:**
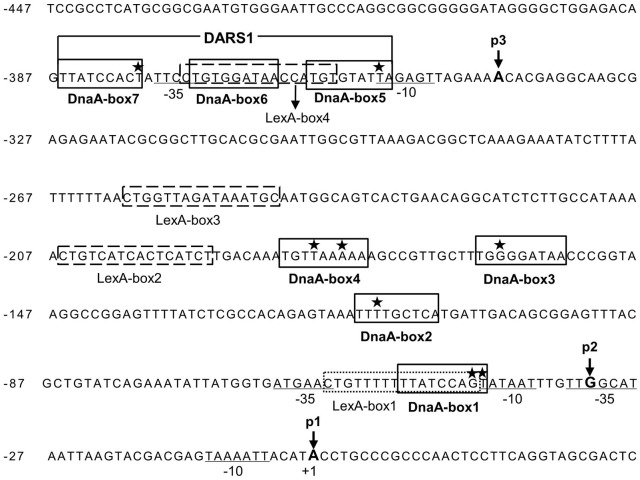
The *uvrB* promoter cluster contains three promoters, seven potential DnaA-boxes and four LexA-boxes. Seven potential DnaA binding sites (DnaA-box1, 2, 3, 4, 5, 6, and 7) in the *uvrB* promoter region are boxed and labeled. DARS1 site consists of DnaA-box 5, 6, and 7. The stars represent the mismatched nucleotides to the consensus DnaA-box (TTA/TTNCACA). The potential LexA-boxes are boxed with a dashed line and labeled. The –35 and –10 sites of the promoters are underlined and labeled. The positions of nucleotides at the transcriptional starting site from promoter *uvrB*p1, p2 and p3 are indicated by p1, p2 and p3 with arrow, respectively, while p1 is numbered as +1 (Sancar et al., 1982; Arikan et al., 1986). The letters in bold indicate the transcriptional starting sites (+1) from different promoters.

In order to understand how LexA and DnaA function in the control of *uvrB* transcription, we searched for the sequences corresponding to DnaA-boxes and LexA-boxes in the *uvrB* promoter region. The *uvrB* promoter region was previously shown to contain three DnaA-boxes (Fujimitsu et al., 2009) and one LexA-box (Van Den Berg et al., 1985). By our analysis, four additional DnaA-boxes and three potential LexA-boxes were identified in the region (**Figure 2**). All these DnaA-boxes were renamed as DnaA-box1, 2, 3, 4, 5, 6, and 7 from the proximal to the distal site relative to the transcription start site of the *uvrB*p1 promoter (**Figure 4A**). The characterized LexA-boxes were called LexA-box1, 2, 3, and 4, also in the same orientation (**Figure 2**). LexA-box1 is closest to the consensus, having the conserved CTG trimer on the left and the CAG trimer on the right end, with nine ATs out of ten bases between these two trimers. LexA-box2, 3, and 4, however, have a CTG on the left but do not have CAG on the right end, having an AT-rich sequence in the middle. LexA-box1 is located between the -35 and -10 sites of the *uvrB*p2 promoter (**Figure 4A**) (Sancar et al., 1982), overlapping with DnaA-box1. LexA-box2 and 3 are found between the *uvrB*p2 and *uvrB*p3 promoters (**Figure 4A**), and LexA-box4 is found within the DARS1 (Fujimitsu et al., 2009), overlapping with DnaA-box5 and 6 on the *uvrB*p3 promoter (**Figures 2**, **4A**). DnaA-box2, 3 and 4 are located between *uvrB*p2 and *uvrB*p3, and three other DnaA-boxes (DnaA-box5, 6, and 7) are found in the *uvrB*p3 promoter (**Figure 2**), composing the DARS1 region (Fujimitsu et al., 2009). The presence of DnaA-boxes in the *uvrB* promoter region support the idea that DnaA might be directly involved in transcription control of the *uvrB* gene and also suggest a possible cooperative or competitive interaction with LexA via their overlapping binding sites (Van Den Berg et al., 1985).

### DnaA Interferes With Binding of LexA to LexA-Box2 and 3 but Not to LexA-Box1

**FIGURE 3:**
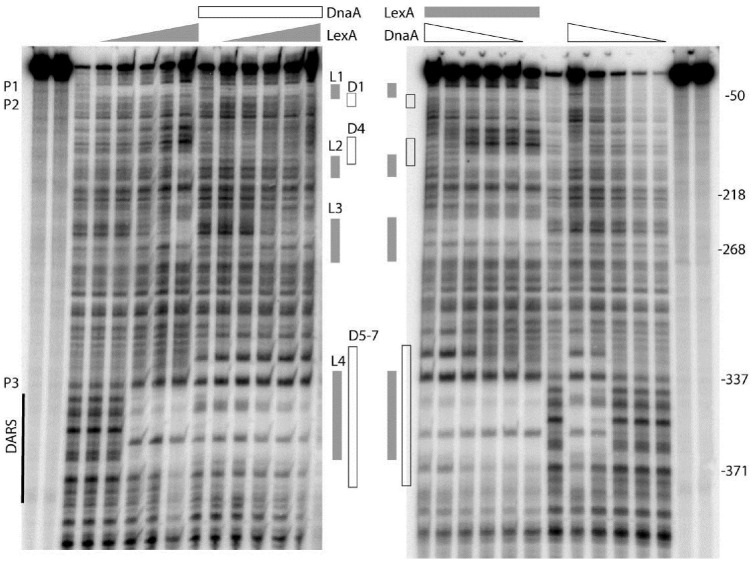
DnaA and LexA protect the *uvrB* promoter region in either competitive or independent manner. The *uvrB* promoter fragment (–513 to +12 relative to the transcription starting site of *uvrB*p1) was generated by PCR using the upstream oligonucleotide labeled with gamma ^32^p-ATP by T4 kinase at the 5′ end. The labeled DNA was either incubated at 37°C with increasing concentrations (50, 100, 200, 300, and 600 nM) of LexA (left) or ATP-DnaA (right) protein. After 10 min incubation of either the ATP-DnaA or the LexA protein with the labeled DNA, the sample had the other protein. The DNA was cleaved by DNase I for 20–30 s after 10 min of incubation with the second protein. The reaction was stopped by addition of an equal volume of formamide gel loading buffer and placed on ice. The gray boxes show the sites protected by the LexA protein. The white boxes correspond to the protection by ATP-DnaA. The protected DnaA-boxes are indicated as D, the LexA-boxes are as L. The DARS site is also shown. P1, P2, and P3 represent promoter *uvrB*p1, *uvrB*p2, *uvrB*p3, respectively. The right and left most two lanes are for ladder.

As described above, we have shown that both DnaA and LexA repress transcription from the *uvrB* promoter cluster, which contains seven potential DnaA-boxes and four LexA-boxes. Now the questions are: (i) do DnaA and/or LexA bind to these potential DnaA-boxes and/or LexA-boxes? (ii) do DnaA and LexA compete for their binding sites in the *uvrB* promoter region? To address these questions, we performed *in vitro* DNase I footprinting experiments and determined the binding patterns of DnaA and LexA to the *uvrB* promoter cluster. A PCR amplified fragment (523 bp) of the *uvrB* promoter cluster was used in these experiments. The DnaA protein was purified as described previously (Olliver et al., 2010) and His_6_-LexA was purified as described in Materials and Methods (Supplementary Figure S1). A protection pattern of the *uvrB* promoter cluster by increasing concentrations of LexA was detected in the presence or absence of DnaA. As shown in **Figure 3**, LexA protections of LexA-box2, 3, and 4 increased as a function of its concentration whereas LexA-box1 became protected at the lowest concentration of LexA. These results are in agreement with the differences in the LexA-box2, 3, and 4 sequences relative to the consensus sequence of LexA-box1. In the presence of DnaA, the LexA protections to LexA-box2, 3, and 4 were weakened or abolished in a DnaA concentration dependent manner while binding to LexA-box1 remained strong (**Figure 3**). For the DARS site, which has the LexA-box4 overlapping with DnaA-box5 and 6, the LexA protection was clear. While the overlap of the protection of the two proteins at the DARS site makes it difficult to determine whether LexA is still bound in the presence of DnaA, the appearance of the DnaA protections at the same concentration as in the absence of LexA suggests that the former can bind even in the presence of the latter and could thus displace it (**Figure 3**). These results suggest that LexA binds to LexA-box1 with high affinity even in the presence of high concentrations of DnaA but not to LexA-box2, 3, and 4. High concentrations of DnaA weaken the binding of LexA to its low affinity boxes. The competition of DnaA for its binding sites with LexA is not necessarily dependent on the fact that DnaA-boxes overlap with the LexA-boxes, possibly due to the ability of ATP-DnaA to form oligomeric structures.

Strong protections at DARS1 were found for ATP-DnaA (**Figure 3**). The DnaA protections of these sites were concentration dependent and such protections were not found to be changed in the presence of LexA. To further understand how DnaA and LexA function in the control of *uvrB* gene expression, we investigated the interaction between DnaA and LexA. The bacterial two-hybrid analysis showed that DnaA did not interact directly with LexA (data not shown).

### The Full Activity of the *uvrB* Promoter Region Requires Both the *uvrB*p1-2 and *uvrB*p3 Promoters

**FIGURE 4:**
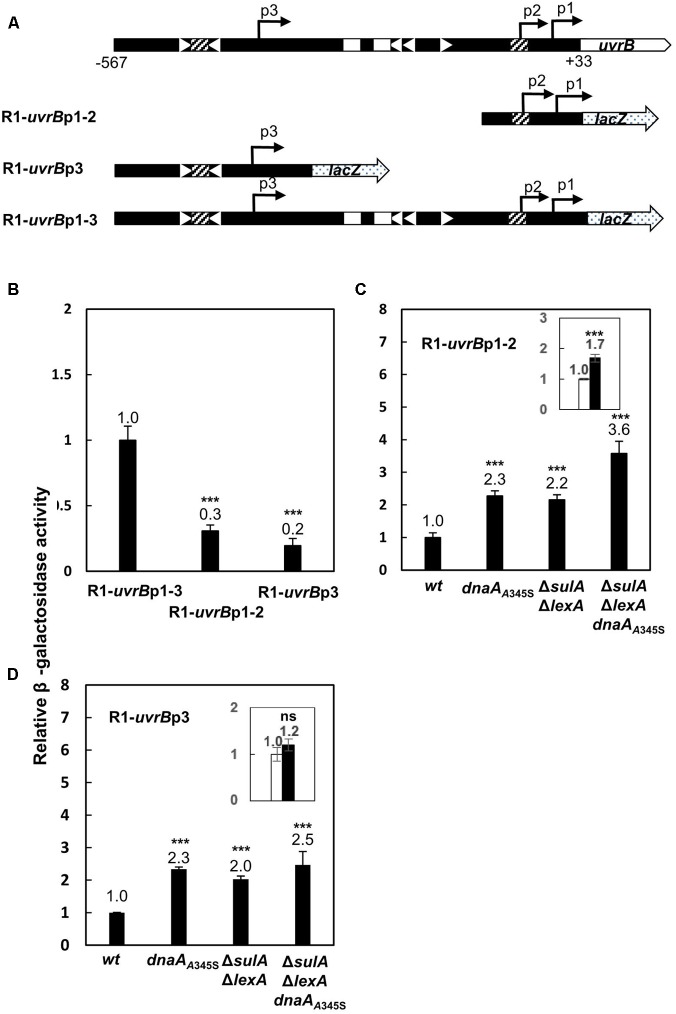
The full activity of the *uvrB* promoter requires the *uvrB*p1-2 and *uvrB*p3 promoters. **(A)** Illustration for insertion of individual *uvrB* promoter in front of the *lacZ* gene into MiniR1 plasmid. Construction of the MiniR1-*uvrB*p1-3-*lacZ* (R1-*uvrB*p1-3), MiniR1-*uvrB*p1-2-*lacZ* (R1-*uvrB*p1-2) or MiniR1-*uvrB*p3-*lacZ* (R1-*uvrB*p3) plasmid was as mentioned in section “Materials and Methods.” Promoter *uvrB*p1-2 contains promoter 1 and 2, ranging from –77 to +33; promoter *uvrB*p3 starts from –567 and ends at –280; Promoter *uvrB*p1-3 consists of promoter 1, 2, and 3, including the region from –567 to +33, the whole cluster of the *uvrB* promoters. The nucleotide positions are as indicated in **Figure 2**. The gray arrows represent the *lacZ* gene. The open rectangles represent LexA-boxes, the open triangles represent DnaA-boxes with orientation, the hatched rectangles represent LexA-boxes overlapping with DnaA-box. The filled arrows indicate positions of the promoters and orientation of transcriptions. The p1, p2, and p3 represent the *uvrB* promoter 1, 2, and 3. The wild-type, *dnaA*_A345S_, Δ*sulA*Δ*lexA* and Δ*sulA*Δ*lexA dnaA*_A345S_ cells were transformed by plasmid R1-*uvrB*p1-3, R1-*uvrB*p1-2, and R1-*uvrB*p3, respectively. The resultant transformants were exponentially grown in ABTGcasa medium at 37°C. Activity of the individual plasmid-borne *uvrB* promoter was measured as the β-galactosidase activity in the cells by Miller method (Miller, 1972). Activity of individual promoter relative to that of R1-*uvrB*p1-3 (229 U) in the wild-type cells is illustrated **(B)**. Relative activity of promoter R1-*uvrB*p1-2 **(C)**, or R1-*uvrB*p3 **(D)** in the *dnaA*_A345S_, Δ*sulA*Δ*lexA* and Δ*sulA*Δ*lexA dnaA*_A345S_ cells compared with that in the wild-type cells (70 U for *uvrB*p1-2 and 44 U for R1-*uvrB*p3) is illustrated. The insets indicate the relative activity of promoters in the Δ*sulA*Δ*lexA dnaA*_A345S_ cells against that in the Δ*sulA*Δ*lexA* cells. The values shown at top of the bars are the average of three individual experiments, and the standard errors are shown. ^∗∗∗^Stands for *P*-value ≤ 0.01, ^∗∗^for 0.01 < *P*-value ≤ 0.05, and ns represents *P*-value > 0.05.

The *uvrB* promoter region has three characterized promoters, namely *uvrB*p1, *uvrB*p2, *uvrB*p3 (Sancar et al., 1982), forming a cluster of *uvrB* promoters (**Figure 4A**). To determine the roles of these different promoters in the transcription control of the *uvrB* gene, each promoter was inserted in front of the *lacZ* gene into the MiniR1 plasmid. MiniR1 is a low copy plasmid, having 1–2 copies per the chromosomal *ter* site (Morigen et al., 2001). The resultant plasmids carry a *uvrB*p1-3-*lacZ* (for short as R1-*uvrB*p1-3), *uvrB*p1-2-*lacZ* (R1-*uvrB*p1-2) or *uvrB*p3-*lacZ* (R1-*uvrB*p3) fusion as illustrated in **Figure 4A**. The R1-*uvrB*p1-3 construct includes all three promoters. Each plasmid was introduced into the wild-type MC4100 cells or the *dnaA*_A345S_, Δ*lexA*Δ*sulA* or Δ*lexA*Δ*sulA dnaA*_A345S_ derivatives (**Figure 4**). Promoter activity was then measured by the β-galactosidase activity assay in exponentially growing cells. Transcription from *uvrB*p1-2 accounted for 30% of the activity of the full-length promoter region while *uvrB*p3 accounted for 20% (**Figure 4B**). The results suggest that full activity of the *uvrB* promoter requires both the *uvrB*p1-2 and *uvrB*p3 promoters.

### Transcription From the Plasmid-Borne *uvrB*p1-2 or *uvrB*p3 Is Repressed by DnaA and LexA Independently

To further clarify the function of DnaA and LexA in the control of the *uvrB*p1-2 or *uvrB*p3 promoter activity, transcription from the plasmid-borne *uvrB*p1-2-*lacZ* construct was measured in the *dnaA*_A345S_, Δ*sulA*Δ*lexA* and Δ*sulA*Δ*lexA dnaA*_A345S_ mutant strains as described above. As shown in **Figure 4C**, transcription in the *dnaA*_A345S_ or Δ*sulA*Δ*lexA* strains was about twofold higher relative to that in the wild-type cells. The transcription level further increased to 3.6-fold of wild type in the Δ*lexA*Δ*sulA dnaA*_A345S_ cells. The increase in transcription from *uvrB*p1-2 in the Δ*sulA*Δ*lexA dnaA*_A345S_ cells compared to that in the Δ*sulA*Δ*lexA* cells was clear (**Figure 4C** inset). These results suggest that *uvrB*p1-2 promoter activity is tightly regulated by both LexA and DnaA, consistent with the presence of LexA- and DnaA-boxes in the promoters. Similarly, transcription from the plasmid-borne *uvrB*p3 was twofold higher in the *dnaA*_A345S_ or Δ*sulA*Δ*lexA* cells compared with that in the wild-type cells, and a slight further increase was also found in the Δ*lexA*Δ*sulA dnaA*_A345S_ mutant (**Figure 4D**) although the increase was not significant (**Figure 4D** inset). The results indicate that both DnaA and LexA repress the *uvrB* expression and function independently.

### Repression of the *uvrB*p3 Promoter Is Largely Dependent on the DnaA-Box6

**FIGURE 5:**
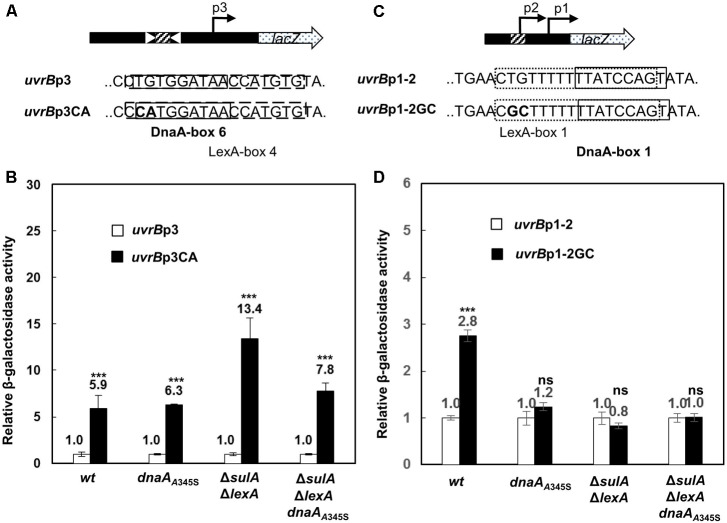
DnaA-box6 in promoter *uvrB*p3 and LexA-box1 in *uvrB*p1-2 are important for control of the promoters. Mutations (TG to CA) in DnaA-box6 on plasmid *uvrB*p3 **(A)** and mutations (TG to GC) in LexA-box1 on plasmid *uvrB*p1-2 **(C)** were introduced, resulting in plasmid p*uvrB*p3CA-*lacZ* (indicated as *uvrB*p3CA for short) **(A)** and p*uvrB*p1-2GC-*lacZ* (as *uvrB*p1-2GC) **(C)**. The wild-type, *dnaA*_A345S_, Δ*sulA*Δ*lexA* and Δ*sulA*Δ*lexA dnaA*_A345S_ cells were transformed with plasmid *uvrB*p3CA and *uvrB*p1-2GC, respectively. Relative activity of the mutated promoter *uvrB*p3CA **(B)** or *uvrB*p1-2GC **(D)** in the wild-type and the mutant cells was compared with that of *uvrB*p3 or *uvrB*p1-2 after measurement of the β-galactosidase activity as mentioned in the legend to **Figure 2**. The open bars stand for *uvrB*p3 or *uvrB*p1-2, the filled bars for *uvrB*p3CA or *uvrB*p1-2GC. The values shown at top of the bars are the average of three individual experiments, and standard errors are shown. ^∗∗∗^Stands for *P*-value ≤ 0.01, ^∗∗^for 0.01 < *P*-value ≤ 0.05, and ns represents *P*-value > 0.05.

The footprinting analysis showed that both DnaA and LexA bound to DnaA-box5, 6, 7 and LexA-box4 of the *uvrB*p3 promoter. To determine the role of such binding sites in the control of the *uvrB*p3 promoter, we mutated the TG in DnaA-box6 to CA on the *uvrB*p3 plasmid (a derivative of pTAC3953 as described in section “Materials and Methods”) by site-directed mutagenesis, leading to the *uvrB*p3CA plasmid (**Figure 5A**). The mutations also changed the LexA-box4 since DnaA-box6 overlaps with LexA-box4 (**Figure 5A**) and destroyed the DARS1 site where ATP-DnaA is formed (Fujimitsu et al., 2009). It was found that transcription from *uvrB*p3CA was 5.9-fold higher relative to that from *uvrB*p3 in the wild-type cells (**Figure 5B**). Compared with *uvrB*p3, transcription from *uvrB*p3CA in the *dnaA*_A345S_ cells was 6.3-fold greater (**Figure 5B**), indicating that mutation of the site can still influence transcription in the absence of full DnaA activity, probably by influencing LexA binding. In the Δ*lexA*Δ*sulA* cells, transcription from *uvrB*p3CA was 13.4-fold higher relative to the activity of *uvrB*p3 (**Figure 5B**). Clearly, in the absence of LexA the mutation of the DnaA-box6 results in a dramatic change in transcription, suggesting that strong repression by the wild type ATP-DnaA is decreased due to the mutations. These same mutations can also impair ATP-DnaA regeneration activity at the DARS1, leading to a decrease in accumulation of ATP-DnaA compared with the wild type plasmid (Fujimitsu et al., 2009).

### LexA-Box1 Is Important for Control of *uvrB*p1-2 Transcription

The *uvrB*p1-2 promoter contains LexA-box1 and DnaA-box1. LexA-box1 remains strongly protected by LexA in the presence of high concentrations of DnaA. To understand the role of LexA-box1 in the regulation of *uvrB*p1-2 transcription, we mutated TG to GC in LexA-box1, resulting in plasmid *uvrB*p1-2GC (**Figure 5C**). These mutations scrambled LexA-box1 since the conserved trimer CTG on the left of LexA-box1 is destroyed. Transcription from *uvrB*p1-2GC was about threefold higher of that from *uvrB*p1-2 in the wild-type cells (**Figure 5D**), indicating that LexA-box1 is important for the control of promoter activity. This result also suggests that binding of LexA to the mutated LexA-box1GC is weakened. However, transcription from *uvrB*p1-2GC did not change relative to that from *uvrB*p1-2 in the *dnaA*_A345S_, Δ*lexA*Δ*sulA* and Δ*lexA*Δ*sulA dnaA*_A345S_ cells (**Figure 5D**). The results indicate that the loss of repression in the mutant strains is thus the same in the presence or absence of the LexA-box1 mutation. This is expected in the case of the Δ*lexA*Δ*sulA* strain, however, it is surprising in the *dnaA*_A345S_ and Δ*lexA*Δ*sulA dnaA*_A345S_ cells since the LexA-box1 mutation should not influence binding by DnaA and a further increase in expression would be expected when DnaA is mutated. It is thus possible that this change in DNA sequence might also affect DnaA oligomerization or RNAP binding.

### UV Irradiation Increases Transcription of the *uvrB* Gene

**FIGURE 6:**
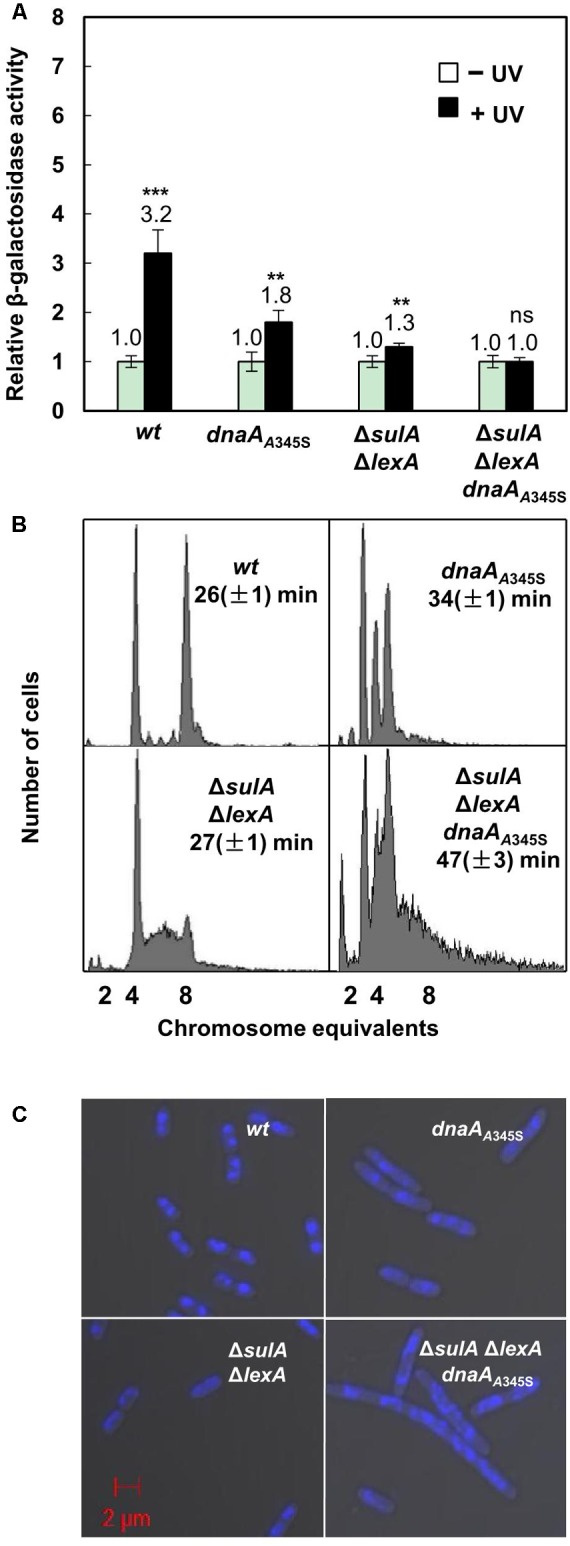
The simultaneous absence of LexA- and DnaA-repression leads to formation of elongated cells with incomplete replication and aberrant nucleoids. **(A)** Exponentially growing wild-type and mutant cells mentioned in the legend to **Figure 1A** except Δ*sulA* in ABTGcasa medium at 37°C were treated with UV (50 J/M^2^) at OD_450_ = 0.08. The expression of the *uvrB* gene was assayed as the β-galactosidase activity described in the legend to **Figure 1**. The filled bars represent expression after UV treatment relative to that from non-treated cells as indicated by the open bars. The values shown at top of the bars are the average of three individual experiments, and the standard errors are shown. ^∗∗∗^Stands for *P*-value ≤ 0.01, ^∗∗^for 0.01 < *P*-value ≤ 0.05, and ns represents *P*-value > 0.05. **(B)** Exponentially growing cells were treated for 4–5 generations with rifampicin and cephalexin to inhibit both initiation of replication and cell division but allowing ongoing replication finish. Then cells were analyzed by flow cytometer after staining with Hoechst 33258 for 30 min. The *X*-axis indicates chromosome equivalents per cell, the *Y*-axis represents the number of cells measured. Each measurement includes 10000 cells. The doubling time and genotype of the cells are shown. **(C)** Exponentially growing cells were harvested and fixed in 70% ethanol. Cells after staining in Hoechst 33258 for 30 min were visualized by Zeiss LSM710 confocal microscope as described in section “Materials and Methods.” The blue structures indicate nucleoids and the red scale bar represents 2 μm.

We found that transcription of the chromosomal *uvrB* gene was increased 3.2-fold in the *uvrB-lacZ* strain after UV irradiation (**Figure 6A**) in the wild-type cells. The level of expression did not significantly change in the Δ*lexA*Δ*sulA* cells after UV irradiation (**Figure 6A**). These results confirm that the *uvrB* gene is one of the SOS genes which are regulated by the LexA protein (Howard-Flanders et al., 1966), responding to UV-induced DNA damage. Interestingly, *uvrB* gene transcription increased less, 1.8-fold, in the *dnaA*_A345S_ cells indicating that DnaA’s decreased repressor activity results in a decreased change in gene expression in the presence of LexA. This is not the case in the Δ*lexA*Δ*sulA dnaA*_A345S_ cells after UV treatment (**Figure 6A**), indicating that LexA is required for UV-dependent SOS induction but not DnaA. These results are consistent with the need of both DnaA and LexA to maintain a high level of repression in the absence of UV treatment.

### The Simultaneous Absence of Both LexA- and DnaA-Dependent Repression on Transcription Results in Elongated Cells With Incomplete DNA Replication

To clarify the role of DnaA-dependent repression on SOS-response genes and its link with DNA replication, we compared nucleoids and cell size in the *dnaA*_A345S_, Δ*lexA*Δ*sulA* and Δ*lexA*Δ*sulA dnaA*_A345S_ cells with that of the wild-type cells. Flow cytometry analysis showed that the wild-type cells had four or eight fully replicated chromosomes, after rifampicin and cephalexin treatment (**Figure 6B**). The DNA histogram of *dnaA*_A345S_ showed well-separated two-, three- and four-chromosome peaks (**Figure 6B**), indicating that the mutant cells contain fully replicated chromosomes although initiation of replication is asynchronous (Gon et al., 2006). However, the Δ*lexA*Δ*sulA* cell culture showed that a portion of the cells contained a DNA amount between four- and eight-chromosome after rifampicin and cephalexin treatment, indicating that a portion of Δ*lexA*Δ*sulA* cells has incomplete replication, probably due to overexpression of DNA repair proteins slowing down the replication forks (**Figure 6B**). The phenotype of incomplete replication was worsened in the Δ*lexA*Δ*sulA dnaA*_A345S_ cells. Some cells had only one-chromosome while other cells had more DNA than eight-chromosome equivalents (**Figure 6B**). These results underline the need for the wild-type DnaA to control both initiation of DNA replication and gene expression when the SOS response is activated. These results suggest that the Δ*lexA*Δ*sulA dnaA*_A345S_ cells have more serious DNA damage, even in the absence of UV irradiation, since severe incomplete replication can be caused from lack of replication elongation or/and partial degradation of the DNA (Skarstad and Boye, 1993; Morigen et al., 2003).

Exponentially growing cells were fixed in 70% ethanol and visualized under Zeiss LSM710 Confocal microscope. As shown in **Figures 6B,C**, both the wild-type and Δ*lexA*Δ*sulA* cells were 2.4 ∼ 3.0 μm in length with a similar doubling time of 26 ∼ 27 min, containing mostly well-compacted one or two nucleoids. The *dnaA*_A345S_ cells were about 4.5 μm in length with a doubling time of 34 min and more nucleoids per cell. The Δ*lexA*Δ*sulA dnaA*_A345S_ cells were further elongated (about 5.5 μm) with a slower growth, and their multi-nucleoids were not well-compacted (**Figures 6B,C** and Supplementary Figure S2). These results together suggest that the simultaneous absence of both LexA- and DnaA-dependent repression of gene transcription results in production of elongated cells with incomplete replication of DNA, aberrant nucleoids and slower growth. It is likely that DnaA-dependent repression of gene transcription during the SOS response is required to prevent over-expression of the SOS regulon genes to maintain genome integrity.

### Transcriptions of the *recN* and *dinJ* Genes Are Also Repressed by Both DnaA and LexA

**FIGURE 7:**
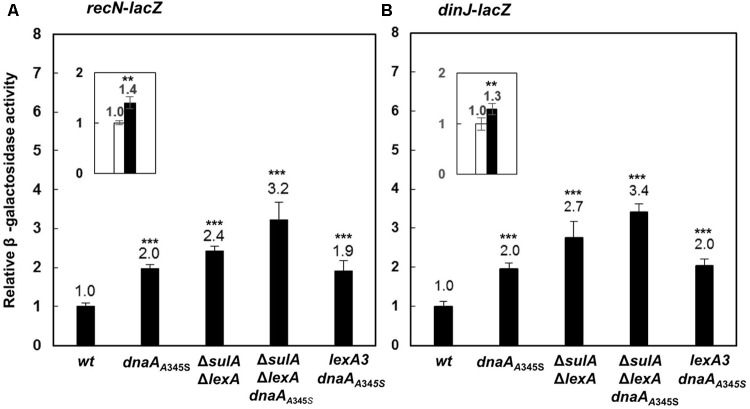
The DnaA and LexA proteins reppress expression of the *recN* or *dinJ* gene independently. The wild-type, *dnaA*_A345S_, Δ*sulA*Δ*lexA*, Δ*sulA*Δ*lexA dnaA*_A345S_ and *lexA*3 *dnaA*_A345S_ cells carrying *recN*-*lacZ* **(A)** or *dinJ*-*lacZ* **(B)** fusion on their chromosomes were exponentially grown in ABTGcasa medium at 37°C. The β-galactosidase activity from *recN*-*lacZ* (13 U for wild-type) **(A)** or *dinJ*-*lacZ* (13 U for wild-type) **(B)** fusion in the cells was determined as described in the legend to **Figure 1**. The insets indicate the relative expression of *renN-lacZ* or *dinJ*-*lacZ* in the Δ*sulA* Δ*lexA dnaA_A345S_* cells against that in the Δ*sulA*Δ*lexA* cells. The values shown at top of the bars are the average of three individual experiments, and the standard errors are shown. ^∗∗∗^Stands *P*-value ≤ 0.01, ^∗∗^ for 0.01 < *P*-value ≤ 0.05, ns represents *P*-value > 0.05.

To test whether DnaA is also involved in regulation of other SOS regulon genes (Finch et al., 1985; Ruangprasert et al., 2014), we searched for DnaA-box and LexA-box in the *dinJ* and *recN* genes and found that both genes had LexA-boxes and a DnaA-box around the transcription start sites as shown in Supplementary Figure S3. We then inserted the *lacZ* reporter gene downstream of the chromosomal *recN* or *dinJ* genes, resulting in *recN-lacZ* or *dinJ-lacZ* derivatives of the MC4100 strain. The *recN-lacZ* or *dinJ-lacZ* allele was transferred to the *dnaA*_A345S_, Δ*lexA*Δ*sulA*, Δ*lexA*Δ*sulA dnaA*_A345S_ and *lexA*3 cells. As shown in **Figures 7A,B**, transcriptions of both the *recN* and *dinJ* genes were about 2 ∼ 3-fold higher in the *dnaA*_A345S_, Δ*lexA*Δ*sulA* and *lexA*3 *dnaA*_A345S_ cells, respectively, indicating that transcription of *recN* or *dinJ* is repressed by both DnaA and LexA, and that the DnaA- or LexA-dependent repression of gene expression is independent. As expected, the increase in transcription of *recN* or *dinJ* was strengthened in the Δ*lexA*Δ*sulA dnaA*_A345S_ cells and it was significant relative to that in the Δ*sulA*Δ*lexA* cells (**Figure 7** insets), suggesting that repression from DnaA and LexA is additive. The observation is supported by the presence of an overlapping LexA-box with a DnaA-box in the *recN* or *dinJ* promoter (Fernandez De Henestrosa et al., 2000). We conclude that DnaA is, indeed, involved in control of other genes of the SOS regulon.

## Discussion

The UvrB protein is a very important protein in the response to DNA damage in prokaryotic cells, performing NER with UvrA and UvrC (Truglio et al., 2006). UvrB paralogs have been found in all organisms (Sancar and Reardon, 2004) and the NER repair system plays a central role in maintaining genome integrity. Defects in NER cause several lines of diseases in humans including skin cancers (Lehmann, 2003). However, the control mechanism of *uvrB* gene expression remains elusive. The results presented here show that the DNA replication initiator, the DnaA protein, and the SOS regulator LexA, regulate the expression of the *uvrB* gene by interacting with the *uvrB* promoter region. The regulation mode by both DnaA and LexA applies to the expression control of several SOS genes, and may be conserved in Gram-negative bacteria. However, this hypothesis requires further experiments to be confirmed.

### DnaA and LexA Regulate Transcription of the *uvrB* Gene By Binding to Their Specific Sites

We have shown that transcription from the *uvrB* promoters is repressed by the presence of both the wild-type LexA and DnaA proteins (**Figure 1A**). The full activity of the *uvrB* promoter cluster requires both *uvrB*p1-2 and *uvrB*p3 promoters and is repressed by both DnaA and LexA in an additive manner (**Figures 4B–D**). The DNase I footprinting experiments show that both proteins bind to the promoter region and that LexA-box1 in *uvrBp*1-2 is the strongest LexA-box from which LexA is not easily displaced by increasing amounts of ATP-DnaA, unlike what is observed at the other LexA-box in this region (**Figure 3**). LexA-box1 has a typical consensus sequence containing both CTG and CAG trimers at the two ends (Walker, 1984; Fernandez De Henestrosa et al., 2000; Wade et al., 2005) while LexA-box2, 3, and 4 do not. Mutations of LexA-box1 and DnaA-box6 in fact show a strong effect on promoter activity *in vivo* (**Figure 5**). The results indicate that binding of LexA or DnaA to LexA-boxes or DnaA-boxes in the *uvrB* promoter region results in a direct control of promoter activity.

The *uvrB*p3 promoter is interesting because it completely overlaps with the DARS1 sequence. While the DARS1 sequence is not essential, in its absence initiation of DNA replication occurs at an increased cell mass (Fujimitsu et al., 2009). Binding of LexA to DARS1 and RNA polymerase to the *uvrB*p3 promoter could both compete with DnaA binding and thus interfere with the regeneration of ATP-DnaA both in the absence (intact LexA) and the presence (RNAP binding) of the SOS response. This can explain the increased loss of repression by the DnaA-box6 mutation in the Δ*lexA*Δ*sulA* strain (**Figure 5B**).

### DnaA and LexA May Coordinate DNA Replication With DNA Repair

LexA-dependent regulation of DARS1 activity is only one of several processes resulting in an increased ATP-DnaA to ADP-DnaA ratio following DNA damage. Upon prolonged stress, fork stalling and blockage of DNA replication, ATP-DnaA accumulates in the cell (Kurokawa et al., 1999). Hydrolysis of the ATP bound to DnaA is mediated by the RIDA process, which requires ongoing DNA replication (Katayama et al., 1998). When the DNA replication forks stall in the presence of DNA damage ATP-DnaA can thus accumulate in the cell and bind to the other sites on the genome. The longer DNA replication has been stalled, the more ATP-DnaA has accumulated in the cell. This would ensure that the expression of DNA repair proteins by the SOS response is limited when the DNA replication forks have stalled and there is less DNA per cell. Furthermore, expression of the *dnaA* gene is induced by DNA damage in a RecA and LexA-dependent manner despite the absence of a LexA-box at the *dnaA* promoter region (Quinones et al., 1991). In these conditions SeqA has been shown to play a key role in cell survival, possibly by limiting over-initiation of DNA replication by increased amounts of ATP-DnaA in the presence of stalled replication forks (Sutera and Lovett, 2006). The increase in ATP-DnaA by decreased RIDA, increased gene expression and increased DARS1 activity occur at the same time as loss of repression by LexA. This may appear to be inconsistent, since they both repress gene expression at several shared targets. However, it is possible that upon DNA damage LexA cleavage results in a rapid loss of repression while the increase in ATP-DnaA occurs with a time delay, due in part to protein synthesis, resulting in a pulse of transcription activity, which, however, is proportional to the number of replication forks, due the amount of ATP-DnaA-dependent repression observed in the absence of DNA damage. It appears to be a situation similar to the hyperinitiation stress observed during oxidative damage (Charbon et al., 2014), but as a sensible response to the problem.

### DnaA-Dependent Transcription Repression During the SOS Response Is Required for Maintaining Genome Integrity: A Model for Control of the SOS Regulon by LexA and DnaA

**FIGURE 8:**
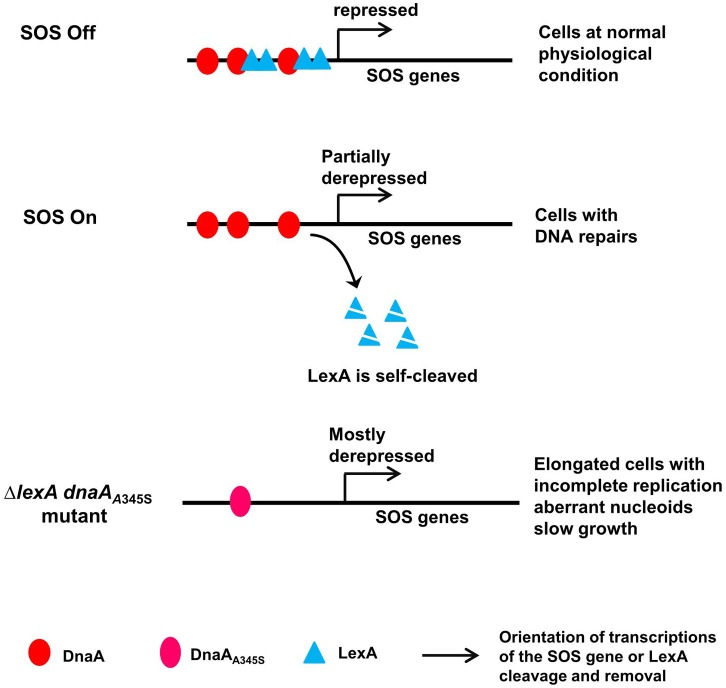
A model for control of the SOS regulon by DnaA and LexA. In the proposed model, both DnaA and LexA repress expression of the SOS genes when SOS is off. When SOS is on, LexA is self-cleaved, but DnaA-repression still works, leading to partial expression of the SOS genes and consequent cells with normal nucleoids and growth. In the simultaneous absence of LexA- and DnaA-repression, the SOS genes are mostly derepressed, forming elongated cells with incomplete replication, aberrant nucleoids and slow growth. The red ovals represent the DnaA protein, the cyan triangles represent the LexA protein, the pink oval represents the DnaA_A345S_ mutant protein, arrows indicate orientation of transcriptions of the SOS gene or LexA cleavage and removal as indicated.

As a summary, we propose a model for the control of the SOS regulon by DnaA and LexA (**Figure 8**). Both DnaA and LexA repress expression of the SOS genes when the SOS response is off. When the SOS response is triggered due to DNA damage, LexA is self-cleaved (Little et al., 1980), consequently LexA-repression is removed to derepress expression of the SOS genes to repair the DNA damage (Sancar et al., 1982). During the SOS response, DnaA-dependent repression still works and largely limits the expression of the SOS genes, resulting in cells with normal nucleoids and growth rate but minor incomplete replication. The latter can be the indication of the DNA repair process since it includes controlled nicking and digestions of the DNA. The simultaneous absence of DnaA- and LexA-repression leads to cell elongation with serious incomplete replication, uncompacted nucleoids and slow growth (**Figures 6B,C**), possibly as a result of a further increase in expression of SOS dependent genes (**Figures 1A**, **7A,B**). Obviously, the high level expression of the SOS genes is harmful, causing physiological problems in different cell processes. Among these problems, incomplete DNA replication can be caused by either lack of replication elongation or partial degradation of the DNA (Skarstad and Boye, 1993; Morigen et al., 2003) as a result of DNA damage. It is reasonable to consider that an excess of DNA repair proteins might “repair” DNA regions where the repairs are unwanted, resulting in DNA damage. Indeed, for example overproduction of DinB has been shown to be lethal (Margara et al., 2016). It is likely that DnaA-dependent repression of the transcription of SOS genes during the SOS response is required to prevent over-expression of the SOS genes to maintain genome integrity.

Interestingly, 13 DnaA-boxes are found in potential LexA-boxes on the *E. coli* chromosomes (Fernandez De Henestrosa et al., 2000). In further analysis, the overlapping LexA-box with a DnaA-box in the *uvrB* or *recN* promoter was found in several Gram-negative bacteria including *Salmonella typhimurium*, *Serratia marcescens*, *Citrobacter rodentium*, *Klebsiella pneumoniae* and *Yersinia enterocolitica* (Supplementary Table S1). These results suggest that DnaA is likely involved in regulation of the SOS regulon, reducing expression of the SOS genes during the SOS response in a manner that is coupled with DNA replication. The control mode may be conserved within Gram-negative bacteria.

## Author Contributions

M, W, and BS conceived and designed the experiments. W, G, EB, SW, and HS performed the experiments. M, W, BS, LF, and YS analyzed the data. M and BS provided reagents. M, W, and BS wrote the manuscript. All authors read the manuscript.

## Conflict of Interest Statement

The authors declare that the research was conducted in the absence of any commercial or financial relationships that could be construed as a potential conflict of interest.

## Abbreviations

ADP/ATP-DnaA: ADP or ATP binds DnaA
*bla*, ampicillin resistance gene, *cat*, chloramphenicol resistance gene, DARS: DnaA reactivation site
DnaA-box: DnaA binding site
FRT: FLP recognition target
LexA-box: LexA binding site
*neo*, kanamycin resistance gene, NER: nucleotide excision repair
RIDA: regulatory inactivation of DnaA
ssDNA: single strand DNA
*tet*: tetracycline resistant gene.
