# Supplementary material for: DnaA and LexA Proteins Regulate Transcription of the uvrB Gene in Escherichia coli: The Role of DnaA in the Control of the SOS Regulon
Source: Front Microbiol. 2018 Jun 18;9:1212. doi: 10.3389/fmicb.2018.01212 (PMC6015884; doi:10.3389/fmicb.2018.01212)
Supplement: Supplementary file 1 [file Presentation_1.PDF]

Supplementary Materials

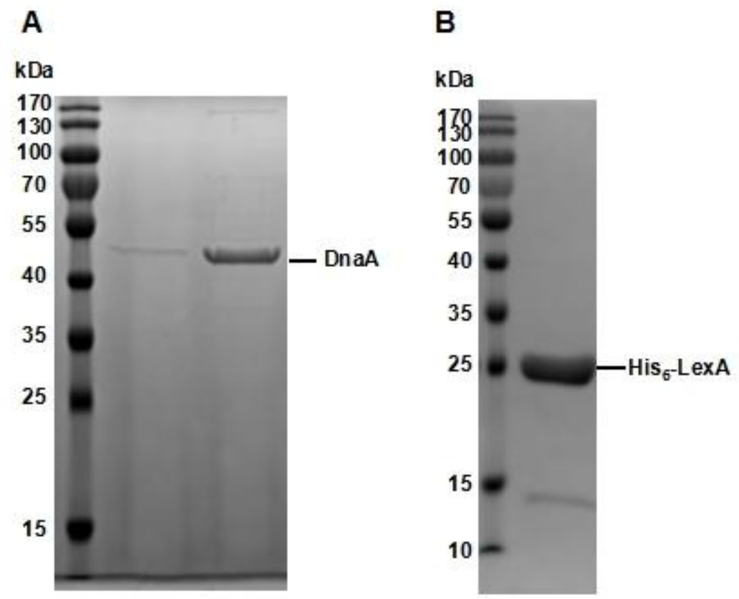

FIG S1 Purification of DnaA and LexA proteins. (A) 52 kDa non-tagged DnaA was purified as described previously (Olliver et al., 2010), shown by INSTANT BLUE staining on SDS/PAGE gel. (B) His-tagged LexA (His6-LexA) protein purified from BL21(DE3) was migrated as a ~ 25kDa protein on SDS/PAGE gel.

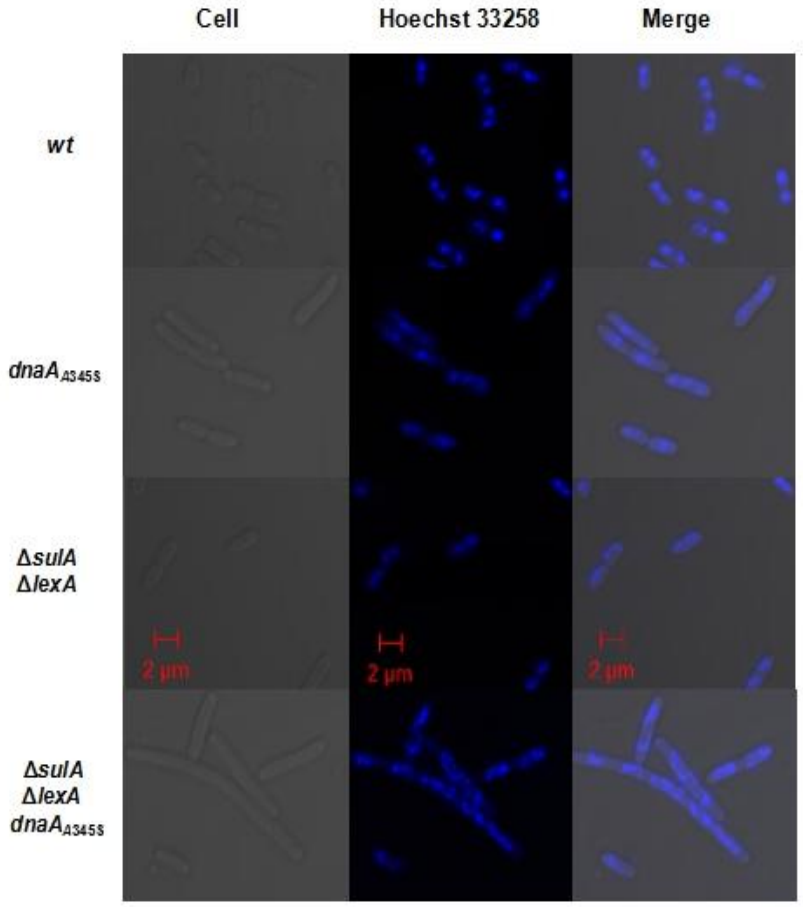

FIG S2 The simultaneous absence of LexA- and DnaA-repression leads to formation of elongated cells with aberrant nucleoids. Exponentially growing cells were harvested and fixed in 70% ethanol. Cells after staining in Hoechst 33258 for 30 min were visualized by Zeiss LSM710 confocal microscope as described in Materials and Methods. The blue structures indicate nucleoids and the red scale bar represents 2 μm.

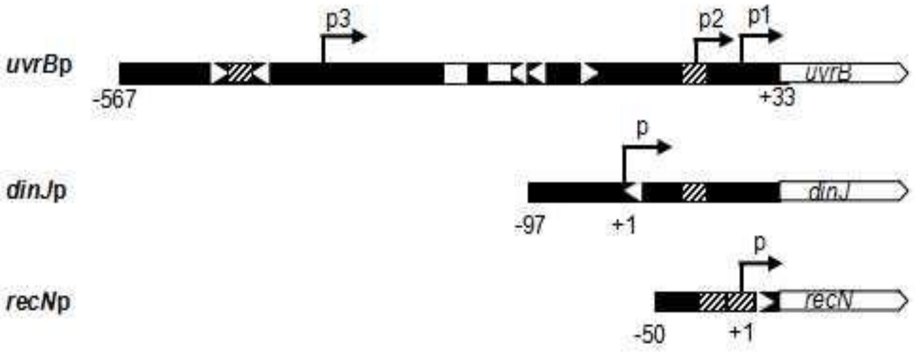

FIG S3 Distribution of DnaA-boxes and LexA-boxes in uvrB promoter and dinJ and recN genes. The open rectangles represent LexA-boxes, the open triangles represent DnaA-boxes with orientation, the hatched rectangles represent LexA-boxes overlapping with DnaA-box. The filled arrows indicate positions of the promoters and orientation of transcriptions.

Table S1. Potential LexA-boxes overlap with DnaA-box in the *uvrB* and *recN* promoters in several gram-negative bacteria.

Species	*uvrB*	*recN*
Escherichia coli	ACTGTTTTTTTTATCCAGT	TACTGTACACAATAACAGTA<br>TACTGTATATAAAACCAGTT
Salmonella typhimurium	GGCAATATTCACCGTCGAG	TACTGTATAAAAAACCAGTT<br>TACTGTATTTAATTACAGTC
Serratia marcescens	AGCTGGTTTTATATCCAGTA	TACTGTATATAAAACCAGTT
Citrobacter rodentium	TACTGTTTTTTCATCCAG	TACTGTATAAAAACCAGTT
Klebsiella pneumoniae	CACTGTTTAAATATCCAGTA	TACTGGATAAAAAACCAGTC
Yersinia enterocolitica	AGCTGGTTTTATATCCAGTA	Not Found

The sequences are LexA-boxes in which DnaA-boxes are underlined.

Table S2. Primers used

ID No.	Sequence(5'---3')	Usage
48	CAGCTGCGTGAGCTGTTTATCGCGGCATCGTAACAGGATAGCGAGTGTAGGCTGGAGCTGCTTC	To construct the *uvrB*-*lacZ* fusion on chromosome with 49
49	TAAGCGTAGCGCATCAGGCTGTTTTCCGTTTGTCATCAGTCTTCTCATATGAATATCCTCCTTAG	To construct the *uvrB*-*lacZ* fusion on chromosome with 48
51	CGCCGCAAGGCTTGAACAAG	To test construction of the *uvrB*-*lacZ* fusion on chromosome with 49
54	GGATCCCATAAACCTTGCCTTGTTGTAG (*Bam*HI)	To fuse the *uvrB*p1-3 or *uvrB*p3 promoter to *lacZ* on pTAC3953 with 57 or 71
57	AAGCTTGAGTCGCTACCTGAAGGAG (*Hin*dIII)	To fuse the *uvrB*p1-3 promoter to *lacZ* on pTAC3953 with 54
71	AAGCTTCTTTGAGCCGTCTTTAACGC (*Hin*dIII)	To fuse the *uvrB*p3 promoter to *lacZ* on pTAC3953 with 54
79	CGGCGGATCCAAATATTATGGTGATGAAC (*Bam*HI)	To fuse the *uvrB*p1-2 promoter to *lacZ* on pTAC3953 with 57
578	CATGCCATGGGCAAAGCGTTAACGGCCAGG(*Nco*I)	To construct plasmid pET28a-his$_6$-*lexA* with 579
579	CCGCTCGAGCAGCCAGTCGCCGTTG (*Xho*I)	To construct plasmid pET28a-his$_6$-*lexA* with 578
582	CTCACAGCATAACTGTATATACACCCAGGGGGCGGAGTGTAGGCTGGAGCTGCTTC	To delete the *lexA* gene from chromosome with 583
583	CGCGACGCCAGGCGGCATCGCGGTCTCAGAGATATGCATATGAATATCCTCCTTAG	To delete the *lexA* gene from chromosome with 582
585	GGCTCTGAATACCATGAGC	To test the *lexA* deletion on chromosome with 583
828	TCTCCCGGGGCGTCTTCGATTGACTGC (*Sma*I)	To amplify the *uvrB* promoter region for the footpringting assay with 829
829	CTGTCTAGAGGGCGGGCAGGTATG (*Xba*I)	To amplify the *uvrB* promoter region for the footpringting assay with 828
1037	GGGGTACCCGCCGTTGCTTTGGGGATAACC (*Kpn*I)	To delete a region of -279 to -172 from *uvrB*p1-3 on p*uvrB*p1-3-*lacZ* with 1038
1038	GGGGTACCCGCTTTGAGCCGTCTTTAACGC (*Kpn*I)	To delete a region of -279 to -172 from *uvrB*p1-3 on p*uvrB*p1-3-*lacZ* with 1037
1131	CCCAAGCTTTTATTTTTGACACCAGACCAACTGG (*Hin*dIII)	To amplify the *uvrB*p1-3-*lacZ* fusion fragment with 54
1210	CCACTATTCC**CA**TGGATAACCATG	To replace TG by CA in DnaA-Box6 on p*uvrB*p3-*lacZ* with 1211

1211	**TG**GGAATAGTGGATAACTGTC	To replace TG by CA in DnaA-Box6 on p*uvrB*p3-*lacZ* with 1210
1214	GGTGATGAAC**GC**TTTTTTTATC	To replace TG by GC in LexA-Box1 on p*uvrB*p1-2-*lacZ* with 1215
1215	**GC**GTTCATCACCATAATATTTC	To replace TG by GC in LexA-Box1 on p*uvrB*p1-2-*lacZ* with 1214
1229	GGCGAATGCGAAAGAACTGCTTG CAGCGTAAACTTTTTTCCTGGTGT AGGCTGGAGCTGCTTC	To construct the *recN*-*lacZ* fusion on chromosome with 1230
1230	GCTTTCCGGTCTTACGGCGTTTTG CTGTTTACTCTGACCGTGAAGCAT ATGAATATCCTCCTTAG	To construct the *recN*-*lacZ* fusion on chromosome with 1229
1232	GTGGATGTAGGGATTAGCGG	To test construction of the *recN*-*lacZ* fusion on chromosome with 1230
1235	GGCCAAAGACGCCGATGATTTATT TGATAAATTAGGAATTTAAATATGT GTAGGCTGGAGCTGCTTC	To construct the *dinJ*-*lacZ* fusion on chromosome with 1236
1236	CATCCTTTGAATATTGTCCCGAGTA TTCAATATCCCTTTGAATCCATATG AATATCCTCCTTAG	To construct the *dinJ*-*lacZ* fusion on chromosome with 1235
1238	GCTGACCATCTCTGACCTGG	To test construction of the *dinJ*-*lacZ* fusion on chromosome with 1235
1350	GGAAGATCTTTATTTTTGACACCA GACCAACTGG (*Bgl*II)	To amplify the *uvrB*p1-2-*lacZ* or *uvrB*p3-*lacZ* fragment with 79 or 54

References

Olliver, A., Saggioro, C., Herrick, J., and Sclavi, B. (2010). DnaA-ATP acts as a molecular switch to control levels of ribonucleotide reductase expression in *Escherichia coli*. *Mol. Microbiol.* 76**,** 1555-1571.

Rostas. K., Morton, S.J., Picksley, S.M., and Lloyd, R.G. (1987). Nucleotide sequence and LexA regulation of the *Escherichia coli recN* gene. *Nucleic. Acids. Res.* 15: 5041-5049.
